# Supplementary material for: The Potential of Ensemble‐Based Automated Sleep Staging on Single‐Channel EEG Signal From a Wearable Device
Source: J Sleep Res. 2026 Jan 19;35(4):e70282. doi: 10.1111/jsr.70282 (PMC13357711; doi:10.1111/jsr.70282)
Supplement: Supplementary file 1 — Table S1: Performance metrics (percentage) for each automatic sleep staging algorithm and the ensemble model across all 30,002 pooled epochs, computed with respect to the consensus of two human scorers. Table S2: Pairwise differences (Δ) between sleep staging algorithms for each performance metric. Figure S1: Confusion matrices for each automatic sleep staging algorithm (green colormaps) and the ensemble model (red colormap) at the pooled‐epoch level. Diagonal elements indicate the percentage of correctly classified epochs for each sleep stage (i.e., sensitivity or recall) compared to the consensus of two expert human scorers, while off‐diagonal elements represent misclassifications. The raw number of epochs per cell is reported in parentheses. [file JSR-35-e70282-s001.docx]

**The potential of ensemble-based automated sleep staging on single-channel EEG signal from a wearable device**

***Running title:* Automated sleep staging and wearable EEG**

Federico Salfi^1^*^†^, Domenico Corigliano^1,2†^, Giulia Amicucci^1^, Samantha Mombelli^3^, Aurora D’Atri^1^, John Axelsson^4,5^, and Michele Ferrara^1^*

*^1^Department of Biotechnological and Applied Clinical Sciences, University of L’Aquila, L’Aquila, Italy*

*^2^Department of Psychology, Sapienza University of Rome, Rome, Italy*

*^3^Center for Advanced Research in Sleep Medicine, Research, center of the Centre intégré universitaire de santé et de services sociaux du Nord de l’Île-de-Montréal (Hôpital du Sacré-Coeur de Montréal), Montreal, Canada*

*^4^Department of Clinical Neuroscience, Karolinska Institutet, Stockholm, Sweden*

*^5^Department of Psychology, Stockholm University, Stockholm, Sweden*

^†^Share the first authorship

*Corresponding authors

Dr. Federico Salfi, *Ph.D.*

Department of Biotechnological and Applied Clinical Sciences

University of L'Aquila

Via Vetoio

67100 L’Aquila (AQ)

Italy

[Federico.salfi@univaq.it](mailto:Federico.salfi@univaq.it)

Prof. Michele Ferrara, *Ph.D.*

Department of Biotechnological and Applied Clinical Sciences

University of L'Aquila

Via Vetoio

67100 L’Aquila (AQ)

Italy

[Michele.ferrara@univaq.it](mailto:Michele.ferrara@univaq.it)

**Supplementary material**

**Table S1.** Performance metrics (percentage) for each automatic sleep staging algorithm and the ensembled model across all 30,002 pooled epochs, computed with respect to the consensus of two human scorers.

| **Metric** | **YASA** | **U-Sleep** | **SleepTransformer** | **DeepResNet** | **Ensemble** |
| --- | --- | --- | --- | --- | --- |
| Accuracy (%) | 84.36 | 86.98 | 87.6 | 88.16 | 88.82 |
| Cohen's κ (%) | 78.36 | 81.84 | 82.81 | 83.56 | 84.45 |
| MCC (%) | 78.48 | 82.32 | 82.91 | 83.75 | 84.67 |
| F1 N1 (%) | 34.72 | 53.47 | 49.42 | 55.71 | 53.94 |
| F1 N2 (%) | 84.7 | 86.56 | 86.99 | 87.7 | 88.37 |
| F1 N3 (%) | 89.23 | 85.59 | 88.91 | 88.75 | 89.46 |
| F1 REM (%) | 88.83 | 94.17 | 93.64 | 94.15 | 94.56 |
| F1 Wake (%) | 77.78 | 87.32 | 87.23 | 87.76 | 88.34 |

*Abbreviations:* MCC, Matthews correlation coefficient; N, non-rapid eye movement sleep; REM, rapid eye movement sleep.

**
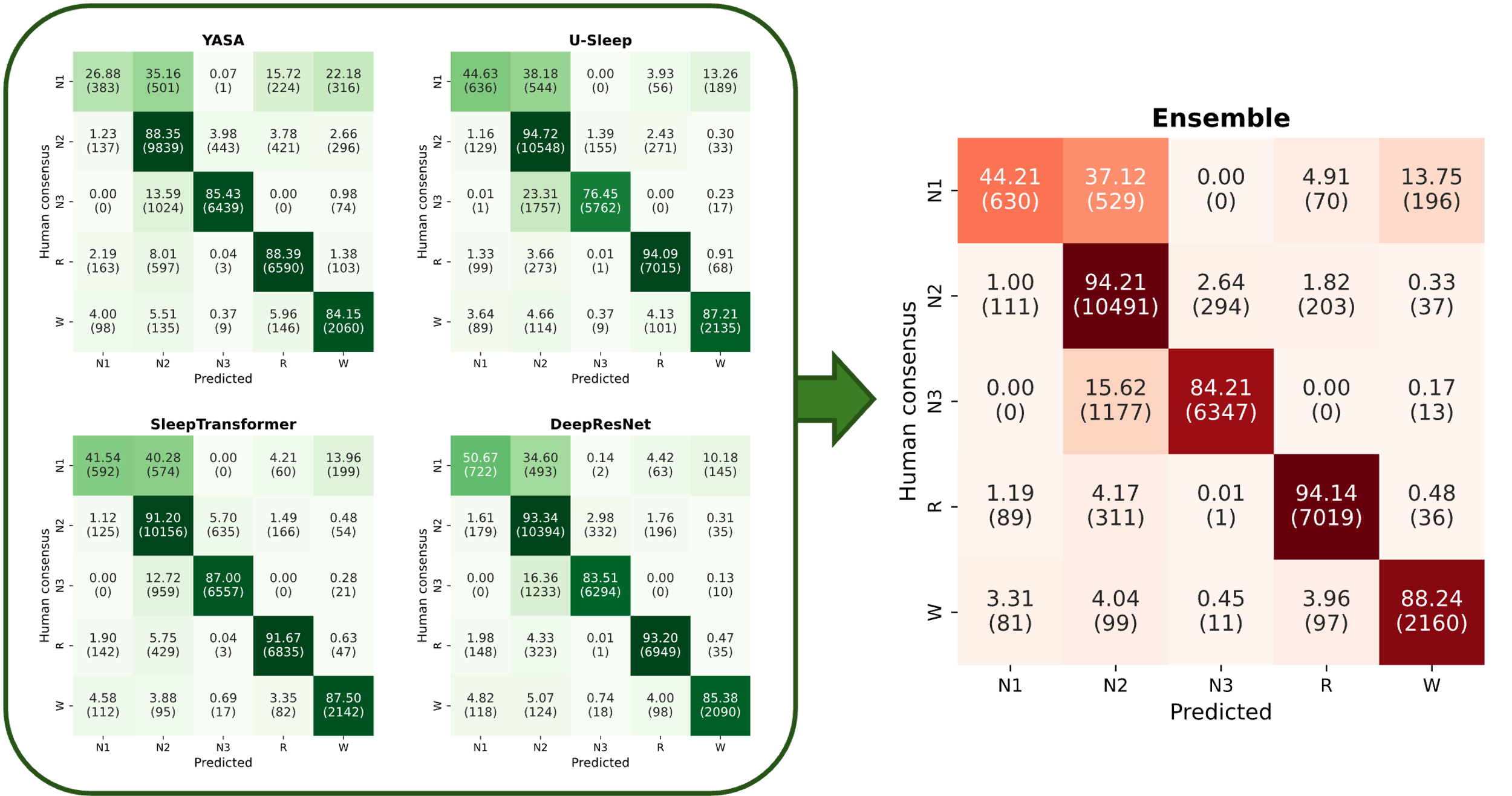
 Figure S1.** Confusion matrices for each automatic sleep staging algorithm (green colormaps) and the ensemble model (red colormap) at the pooled-epoch level. Diagonal elements indicate the percentage of correctly classified epochs for each sleep stage (i.e., sensitivity or recall) compared to the consensus of two expert human scorers, while off-diagonal elements represent misclassifications. The raw number of epochs per cell is reported in parentheses.

*Abbreviations:* N, non-rapid eye movement sleep; REM, rapid eye movement sleep.

**Table S2.** Pairwise differences (Δ) between sleep staging algorithms for each performance metric.

| **Δ Metric** | **YASA**  **vs**  **U-Sleep** | **YASA**  **vs**  **SleepTransformer** | **YASA**  **vs**  **DeepResNet** | **U-Sleep**  **vs**  **SleepTransformer** | **U-Sleep**  **vs**  **DeepResNet** | **SleepTransformer vs**  **DeepResNet** |
| --- | --- | --- | --- | --- | --- | --- |
| Accuracy (%) | −2.77*** | −3.35*** | −4.04*** | −0.58 | −1.27** | −0.69 |
| Cohen's κ (%) | −3.65*** | −4.61*** | −5.53*** | −0.96 | −1.87** | −0.92 |
| MCC (%) | −3.93*** | −4.52*** | −5.47*** | −0.59 | −1.55** | −0.95 |
| F1 N1 (%) | −18.18*** | −14.85*** | −20.73*** | +3.33* | −2.55* | −5.87*** |
| F1 N2 (%) | −1.99** | −2.37** | −3.31*** | −0.39 | −1.32** | −0.93 |
| F1 N3 (%) | +3.60** | +0.54 | +0.56 | −3.06* | −3.05*** | +0.02 |
| F1 REM (%) | −6.04*** | −5.62*** | −6.24*** | +0.41 | −0.21 | −0.62 |
| F1 Wake (%) | −9.71*** | −9.78*** | −9.67*** | −0.07 | +0.04 | +0.11 |

*Abbreviations:* MCC, Matthews correlation coefficient; N, non-rapid eye movement sleep; REM, rapid eye movement sleep.

*Notes:* Negative values indicate lower performance of the first system relative to the second. Asterisks indicate statistically significant differences from paired t-tests (**p* < 0.05, ***p* < 0.01, ****p* < 0.001; row-wise adjusted for multiple comparisons using the Holm method) between autoscoring systems.
